# Modelling Estimates of Norovirus Disease in Patients with Chronic Medical Conditions

**DOI:** 10.1371/journal.pone.0158822

**Published:** 2016-07-20

**Authors:** Thomas Verstraeten, Baoguo Jiang, John G. Weil, Jennifer H. Lin

**Affiliations:** 1 P95 Pharmacovigilance and Epidemiology Services, Leuven, Belgium; 2 Takeda Development Center Americas, Inc., Deerfield, Illinois, United States of America; 3 Takeda Pharmaceuticals International, Zurich, Switzerland; Johns Hopkins Bloomberg School of Public Health, UNITED STATES

## Abstract

**Background:**

The burden of disease due to norovirus infection has been well described in the general United States population, but studies of norovirus occurrence among persons with chronic medical conditions have been limited mostly to the immunocompromised. We assessed the impact of norovirus gastroenteritis on health care utilization in US subjects with a range of chronic medical conditions.

**Methods:**

We performed a retrospective cohort study using MarketScan data from July 2002 to December 2013, comparing the rates of emergency department visits, outpatient visits and hospitalizations among patients with chronic conditions (renal, cardiovascular, respiratory, immunocompromising, gastrointestinal, hepatic/pancreatic and neurological conditions and diabetes) with those in a healthy population. We estimated the rates of these outcomes due to norovirus gastroenteritis using an indirect modelling approach whereby cases of gastroenteritis of unknown cause and not attributed to a range of other causes were assumed to be due to norovirus.

**Results:**

Hospitalization rates for norovirus gastroenteritis were higher in all of the risk groups analyzed compared with data in otherwise healthy subjects, ranging from 3.2 per 10,000 person-years in persons with chronic respiratory conditions, to 23.1 per 10,000 person-years in persons with chronic renal conditions, compared to 2.1 per 10,000 among persons without chronic conditions. Over 51% of all norovirus hospitalizations occurred in the 37% of the population with some form of chronic medical condition. Outpatient visits for norovirus gastroenteritis were also increased in persons with chronic gastrointestinal or immunocompromising conditions.

**Conclusion:**

Norovirus gastroenteritis leads to significantly higher rates of healthcare utilization in patients with a chronic medical condition compared to patients without any such condition.

## Introduction

Norovirus is the most common cause of acute gastroenteritis (AGE) [1]. Transmission may be person-to-person, or via food, water or environmental sources [2,3]. Populations at increased risk of norovirus gastroenteritis (NGE) are the very young, elderly, immunocompromised and those living in closed communities such as long-term care facilities [4,5]. Severe or prolonged symptoms after norovirus infection have been associated with age ≥65 years and the presence of pre-existing medical conditions [6].

As molecular diagnostic techniques capable of identifying norovirus are not in routine use there has been limited direct laboratory-confirmed case evaluation of the NGE burden, indirect modelling techniques that do not require a viral time series based on laboratory confirmed cases have been used recently to assess the NGE burden [7–11].

Modelling studies estimate that in the United States (US) the all-age annual incidence of NGE-associated hospital discharges was 2.4/10,000 population (1996–2007) [10], and that the all-age annual incidence of NGE-associated emergency department (ED) visits and outpatient visits was 13.5/10,000 population and 57.2/10,000 population, respectively (2001–2009) [11]. Norovirus is thus estimated to result in more than 400,000 ED visits, 1.7 million outpatient visits and 70,000 hospitalizations annually in the US [10,11], with higher numbers estimated in epidemic years. These estimates are derived from administrative databases such as the Nationwide Inpatient Sample, and have thus far focused on the general population, not taking into account underlying morbidities. There is evidence that norovirus infections in patients with chronic conditions such as cardiovascular disease, renal transplant recipients and patients on immunosuppressive therapy may lead to severe consequences [6]. To date, no systematic assessment of incidence rates of norovirus disease among patients with chronic medical conditions has been performed.

Based on a similar modelling approach to that used previously to estimate the overall burden of norovirus disease [11], we used insurance claim data to compare the rates of ED visits, outpatient visits and hospitalizations among patients with chronic conditions (renal, cardiovascular respiratory, immunocompromising, gastro-intestinal, hepatic/pancreatic and neurological conditions and diabetes) with those in a healthy population, stratifying by age and chronic condition.

## Methods

### Design and objectives

This was a retrospective cohort study designed to compare episode rates of AGE and NGE in terms of ED visits, outpatient visits and hospitalizations in the patient population with chronic conditions (renal, cardiovascular, respiratory, immunocompromising, gastro-intestinal, hepatic and pancreatic, neurological, and diabetic disorders), against rates in the general population. A secondary study objective was to compare ED visits, outpatient visits and hospitalization episode rates per age category (0–4, 5–17, 18–64, 64–74, 75–84, 85+ years).

### Data source

The Truven Health MarketScan® Commercial Claims and Encounters Database used in this study contains healthcare utilization data for over 140 million individuals in the US enrolled from a variety of public and private healthcare plans since 2001. The study population included all individuals with at least 6 months of previous continuous enrollment record in the database between July 2002 and December 2013.

We extracted all records (diagnosis claims) of ED visits, outpatient visits and hospitalizations between July 2002 until June 2013 with an International Classification of Disease 9^th^ revision (ICD-9) code indicating cause unspecified and cause-specified AGE (Table 1). For cases with multiple AGE-related diagnoses, episodes were considered new if they were at least 14 days apart. The outcomes (ED visits, outpatient visits and hospitalization) were counted separately. Thus, if a patient with a AGE episode treated as an outpatient was later hospitalized and both events were recorded in the database, that patient would be counted once in the outpatient analyses and once in the hospitalization analyses. The sub group of the study population with chronic medical conditions were those with ICD-9 codes indicative of chronic disease that occurred at least twice in 12 months (preceding the AGE code in the AGE cases). The increased risk of severe influenza among persons with chronic conditions has lead to preferential vaccine recommendations in the past [12]. Based on the group of medical conditions listed by the Centers for Disease Control and Prevention as being at high risk for severe influenza [12], we classified patients into eight chronic condition groups using ICD-9 listed disorders which occurred prior to AGE diagnosis (Table 1). Patients were included in one of the eight chronic condition groups when they had a diagnosis of only one of the specified medical conditions. Patients were included in a group of “two or more conditions” when they had diagnosis claims of at least 2 chronic conditions.

**Table 1:** Diagnostic codes used to identify gastroenteritis episodes and chronic medical conditions.

	Description	ICD-9
**Outcome codes**		
Acute gastroenteritis	Cause-unspecified	009.0–009.3, 558.9, 787.91, 008.8 (excluding records with a specific cause in a subsequent diagnostic position)
	Rotavirus	008.61
	Norovirus	008.63
	*Clostridium difficile*	008.45
	Other Bacterial	001.0–00.9, 002.0–002.9, 003.0–003.9, 004.0–004.9, 005.0–005.9, 008.0–008.5
	Parasitic	006.0–006.2, 006.8‐006.9, 007.0–007.9
**Chronic medical conditions**		
Chronic cardiovascular disease	402	Hypertensive heart disease
	404	Hypertensive heart and chronic kidney disease
	410–414	Ischaemic heart disease
	428	Heart failure
	430–438	Cerebrovascular Disease
	997.02	Iatrogenic cerebrovascular haemorrhage or infarction
	V42.1	Heart transplant
	V45.81 -V45.82	Aortacoronary bypass and coronary angioplasty
Chronic renal disease	250.4	Diabetes with renal manifestations
	285.21	Anaemia in chronic kidney disease
	403	Hypertensive chronic kidney disease
	404	Hypertensive heart and chronic kidney disease
	582	Chronic glomerulonephritis
	583	Nephritis and nephropathy not specified as acute or chronic
	584	Acute kidney failure
	585	Chronic kidney disease
	586	Renal failure, unspecified
	588	Disorders resulting from impaired renal function
	792.5	Cloudy aspect haemodialysis
	996.56	Mechanical complication haemodialysis
	996.73	Complication renal dialysis device
	996.81	Complications transplanted kidney
	V42.0	Kidney transplant
	V56	Encounter for dialysis
Chronic respiratory disease	491	Chronic Bronchitis
	492	Emphysema
	493	Asthma
	494	Bronchiectasis
	495	Extrinsic allergic alveolitis
	496	Chronic Airway obstruction, not elsewhere specified
	518.1	Interstitial emphysema
	518.82	Other pulmonary insufficiency, not elsewhere classified
	518.83	Chronic respiratory failure
	518.84	Acute and chronic respiratory failure
	500–508	Pneumoconioses and other lung diseases due to external agents
	V 426	Lung Transplant
Chronic immunocompromising condition	279	Disorders involving the immune mechanism
Diabetes	250.x1, 250.x3	Type 1 DM
	250.x0, 250.x2	Type 2 DM
	357.2	polyneuropathy in diabetes
	362.0	diabetic retinopathy,
	357.2	polyneuropathy in diabetes
	366.41	diabetic cataract
Chronic gastro-intestinal disease	555	Regional enteritis (Crohn)
	556	Ulcerative enterocolitis
	557.1	Chronic vascular insufficiency of intestine
	562	Diverticula of intestine
	564	Functional digestive disorders not elsewhere classified
	569.6	Colostomy and enterostomy complications
	569.82	Ulceration of intestine
	569.83	Perforation of intestine
	579	Intestinal malabsorption
Chronic hepatic and pancreatic disorders	570–577	Chronic hepatic and pancreatic disorders
Chronic neurological disorders	318.1–318.2	Severe and profound intellectual disability
	330–337	Hereditary And Degenerative Diseases Of The Central Nervous System
	340	Multiple sclerosis
	341	Other demyelinating diseases of central nervous system
	342	Hemiplegia and hemiparesis
	343	Infantile cerebral palsy
	344	Other paralytic syndromes
	345	Epilepsy and recurrent seizures
	358	Myoneural disorders
	359	Muscular dystrophies and other myopathies
	438	Late effects of cerebrovascular disease
	756.4	Chondrodystrophy

### Statistical analysis

The diagnostic codes for NGE are infrequently used [10]. We therefore used an indirect method for estimating NGE-associated episodes proposed by Gastañaduy et al [11]. Specifically, the number of cause-unspecified AGE events was first regressed against four pathogen-specified AGE events (i.e., rotavirus, bacterial, *Clostridium difficile*, and parasite-associated events) in each month (Eq 1).

E(cause−unspecifiedAGEij)=α+(β1x N−Rotavirus−eventsi,j)+(β2x N−bacterial−eventsi,j)+(β3x N−C.difficile−eventsi,j)+(β4x N−parasitic−eventsi,j)+ƴx Timei,j (1)

Where,

*α* = intercept,

*β*_*(1–4)*_ = the relative contribution of each of the four specified pathogens,

N-events_*i*,*j*_ = counts of cause-specified events in month *i* of the year *j*, by age and by risk group,

ƴ = the relative contribution of time,

Time_*i*,*j*_ = month *j* in year *i*.

Assuming that any remaining seasonality not explained by the regression model is due to NGE, the number of NGE events in each month was calculated as the difference between predicted cause-unspecified counts and the observed cause-unspecified counts (i.e., the residual of the model). Also assuming that there is a minimum monthly residual for each seasonal year with no NGE events, the estimated number of NGE events was calculated as the difference between the residual of the model and the minimum residual for that seasonal year (from July through June) [11]. Estimates were obtained using Poisson regression with adjustment for overdispersion using the scale of Pearson’s chi-squared statistic. Finally, the medically coded NGE counts were added to the estimated NGE counts each month to get the overall monthly estimates of NGE events. These steps were repeated for each care setting (ED and outpatient visits, and hospitalization) within each risk group and each age group.

The AGE and NGE episode rate was defined as the ratio of the number of events during the study period, divided by the total person-time (i.e., person-month or person-year) of eligible population (or specific patient population) per age group. Person-time was estimated by adding up the number of person-days contributed, per month or per season from July to June of the following year, by each person to each specific age group and each specific risk group. Rates were expressed per 10,000 person-years. We thus estimated the rates of healthcare visits due to AGE and NGE for the overall population, for each of the chronic condition groups, for those who had more than one of these conditions, and for those without any chronic condition. To account for important differences in the age distribution of some of these conditions, we age-standardized all rates to the overall population in the MarketScan database, using the same age categories described above. The 95% confidence intervals (CIs) for the NGE rates were estimated using Monte Carlo simulations accounting for the model uncertainties.

## Results

**Fig 1:**
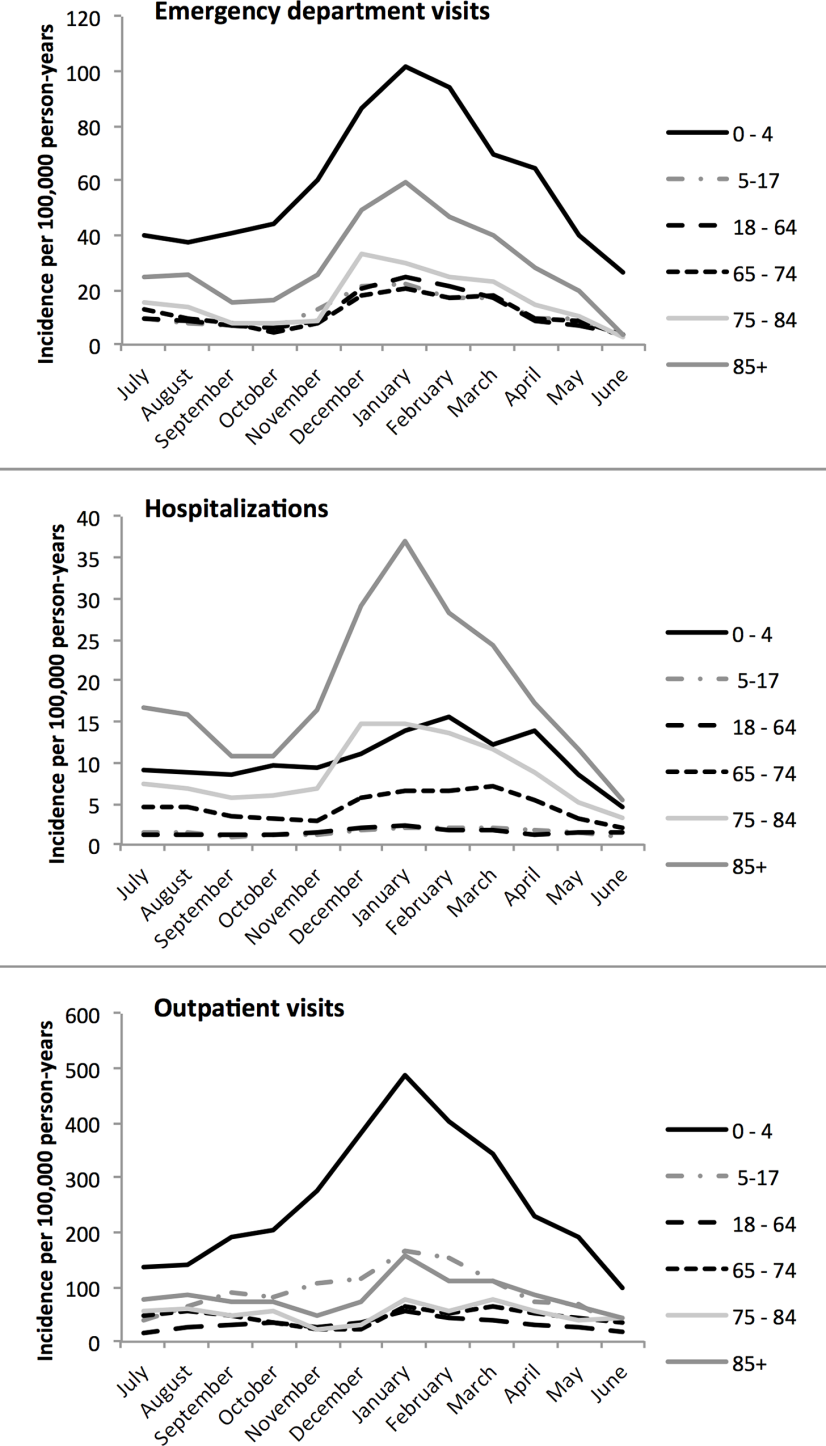
Seasonality of norovirus gastroenteritis healthcare utilization: mean annual incidence (per 100,000 population) by age (2002–2013).

The study population comprised 9,028,466 individuals registered in the MarketScan database, of which 56.4% were female (S1 Table: Demographic features of the study population (MarketScan database, 01 July 2002–30 June 2013)). NGE-attributable healthcare utilization occurred all year-round, but peaked between December and April (Fig 1), particularly in the youngest and eldest age groups.

Utilization rates were rather constant over the years of the study, with some increases in the season 2002–2003 and the seasons 2005–2006 and 2006–2007 (S1 Fig), corresponding to the years with high norovirus circulation [10]. The mean annual rates of ED and outpatient visits attributed to NGE were highest in 0–4 year olds (80.0/10,000 person years and 354.7/10,000 person-years, respectively), followed by 85+ year olds (33.5/10,000 person years and 96.7/10,000 person years, respectively) (Table 2). The mean annual hospitalization rate for NGE was 3.1 per 10,000 person years across all ages and 14.2 per 10,000 person years in 0–4 year olds. The highest NGE-attributable annual hospitalization rate occurred in 85+ year olds (21.3/10,000 person years). The proportion of AGE attributed to norovirus across all age groups was 25.1%, 16.9% and 18.7% for ED visits, outpatient visits and hospitalizations, respectively.

**Table 2:** Number and mean incidence rate (per 10,000 person-years) of all-cause (AGE) and norovirus gastroenteritis (NGE)-attributable emergency department visits, outpatient visits and hospitalizations (2002–2013, MarketScan database).

Age (years)	Emergency department visits				Outpatient visits				Hospitalizations			
	AGE		NGE		AGE		NGE		AGE		NGE	
	N	Rate	N	Rate	N	Rate	N	Rate	N	Rate	N	Rate
0–4	274,783	219.9	91,812	80.0	1,852,298	1471.4	424,119	354.7	37,337	33.7	14,702	14.2
5–17	267,926	49.9	75,440	14.8	1,651,536	312.9	590,128	112.1	27,080	5.5	6,246	1.4
18–64	1,411,636	66.1	310,224	14.7	7,313,701	351.4	797,613	40.4	271,732	13.2	35,580	1.9
65–74	91,692	57.1	19,599	12.9	627,955	399.3	77,279	52.2	51,286	33.4	7,888	5.5
75–84	94,603	94.2	18,364	18.9	544,464	549.3	59,324	62.6	63,771	65.0	9,929	10.5
85+	53,686	123.5	14,045	33.5	250,714	593.9	38,521	96.7	37,302	90.2	8,221	21.3
All ages*	2,194,326	71.2	529,484	17.9	12,240,668	406.1	1,986,984	68.7	488,508	16.6	82,566	3.1

*age standardized rate

### AGE- and NGE-associated episodes in patients with chronic medical conditions

The presence of a chronic condition was identified in 36.5% of the MarketScan population (Table 3). With the exception of immunocompromising conditions which were reported by <1% of the population at any age, the prevalence and type of chronic condition was strongly age-related. Respiratory conditions were most frequent in children and adolescents and decreased with age, whereas the frequency of all other chronic conditions increased with age. Gastrointestinal, and hepatic and pancreatic conditions peaked in 18–64 year olds, diabetes was most frequent in 65–74 year olds, while renal, cardiovascular and neurological conditions all continued to increase with age. Among 65–74 year olds, 76.4% already had at least one chronic medical condition and nearly half (48.9%) had two or more chronic conditions.

**Table 3:** Proportion of the total population found to be free of any chronic medical condition, to have any of the chronic medical conditions included, to have only one chronic medical condition (per group of conditions) and more than one chronic medical condition in the MarketScan population between 2002 and 2013, per age group and overall.

Age (years)	Free of chronic condition	Any chronic condition	Persons with one chronic condition								
			Renal	Cardiovascular	Respiratory	ImmunoC	Gastrointestinal	Hep & Panc	Neurological	Diabetes	≥ 2 conditions
0–4	86.7%	13.3%	0.1%	0.2%	10.4%	0.1%	0.7%	0.1%	0.7%	0.1%	0.9%
5–17	82.5%	17.5%	0.1%	0.1%	11.3%	0.1%	1.9%	0.5%	1.2%	0.5%	1.8%
18–64	60.5%	39.5%	0.5%	2.6%	4.6%	0.1%	7.9%	3.5%	1.3%	4.4%	14.7%
65–74	23.6%	76.4%	0.9%	7.5%	3.5%	0.1%	6.7%	1.9%	1.2%	5.7%	48.9%
75–84	13.8%	86.2%	1.1%	9.4%	2.6%	0.0%	3.9%	1.1%	1.6%	3.2%	63.3%
85+	11.3%	88.7%	1.5%	12.7%	2.0%	0.0%	2.1%	0.7%	2.2%	1.9%	65.5%
All ages	63.5%	36.5%	0.4%	2.5%	6.3%	0.1%	5.6%	2.3%	1.2%	3.1%	14.9%

ImmunoC = immunocompromising condition, Hep & Panc = hepatic and pancreatic condition, ≥ 2 = having at least 2 chronic medical conditions

### AGE and NGE-attributed outpatient and ED visits, and hospitalizations in the overall population and in patients with chronic condition(s)

Mean annual rates of AGE and NGE-attributable outpatient and ED visits in patients with a chronic medical condition were similar to rates in the total population, with the exception of increases in both AGE and NGE-attributable outpatient visits in persons with gastrointestinal conditions, and of NGE-attributable outpatient visits in persons with immunocompromising conditions (Table 4). In contrast, compared with the total population, rates of AGE and NGE-attributable hospitalizations were at least 2-fold higher in patients within all chronic condition groups, except for the respiratory group. Notably, the highest increases for AGE and NGE-attributable hospitalizations were in persons with renal and gastrointestinal conditions, with nearly 8 to more than 10-fold higher rates compared to persons without a chronic condition (Table 4). Persons with two or more chronic conditions were also at increased risk of both AGE and NGE-attributable hospitalizations, with rates roughly halfway between the lower and higher rates observed for the individual chronic conditions. The proportions of AGE attributable to norovirus were generally higher in persons with chronic conditions, particularly for hospitalizations. The highest rates were seen among persons with an immunocompromising or neurological condition in whom the proportion of hospitalizations attributable to norovirus was 68.0% and 56.2%, respectively.

**Table 4:** Mean annual rate (per 10,000 person-years) of all-cause (AGE) and mean annual rate (per 10,000 person-years with 95% confidence intervals) of norovirus gastroenteritis (NGE) emergency department (ED), outpatient visits and hospitalizations in all age-groups: 2002–2013 MarketScan database, age standardized.

	Total population	Free of chronic conditions	Persons with one chronic medical condition								
			Renal	Cardiovascular	Respiratory	ImmunoC	Gastrointestinal	Hep& Panc	Neurological	Diabetes	≥2 conditions
AGE ED visits	71.2	53.3	61.2	37.6	46.5	52.6	67.9	59.6	46.5	52.3	58.0
AGE Outpatient visits	406.1	303.4	266.7	210.4	245.6	382.8	793.5	332.9	245.9	234.4	335.0
AGE Hospitalizations	16.6	6.4	48.1	12.2	8.4	22.8	56.4	25.0	12.1	12.5	38.3
NGE ED visits	17.9	15.5	25.5	14.4	14.7	31.0	21.5	20.9	19.6	21.2(	18.0
	(16.9–20.5)	(13.8–18.1)	(24.2–31.1)	(13.4–18.1)	(13.4–16.8)	(23.6–41.4)	(19.6–24.4)	(19.8–24.7)	(18.2–22.2)	(19.7–24.7)	(17.0–20.8)
NGE Outpatient visits	68.7	60.9	98.4	62.6	55.9	146.4	124.6	88.3	62.2	74.8	67.8
	(63.5–77.2)	(51.9–69.9)	(91.8–111.6)	(58.1–71.1)	(52.0–62.2)	(129.1–166.0)	(115.0–139.7)	(81.9–99.4)	(57.9–69.1)	(69.6–84.3)	(62.7–75.3)
NGE Hospitalizations	3.1	2.1	23.1	4.1	3.2	15.5	16.7	11.1	6.8	5.3	9.8
	(3.0–3.4)	(1.9–2.2)	(21.7–27.7)	(3.7–5.3)	(3.1–3.5)	(11.3–22.8)	(15.-18.7)	(10.2–13.2)	(6.5–7.9)	(4.7–6.5)	(9,3–11.2)

ImmunoC = immunocompromising condition, Hep & Panc = hepatic and pancreatic condition, ≥ 2 conditions = having at least 2 chronic medical conditions

### NGE-attributed outpatient and ED visits and hospitalizations in patients with a chronic condition, by age

**Fig 2:**
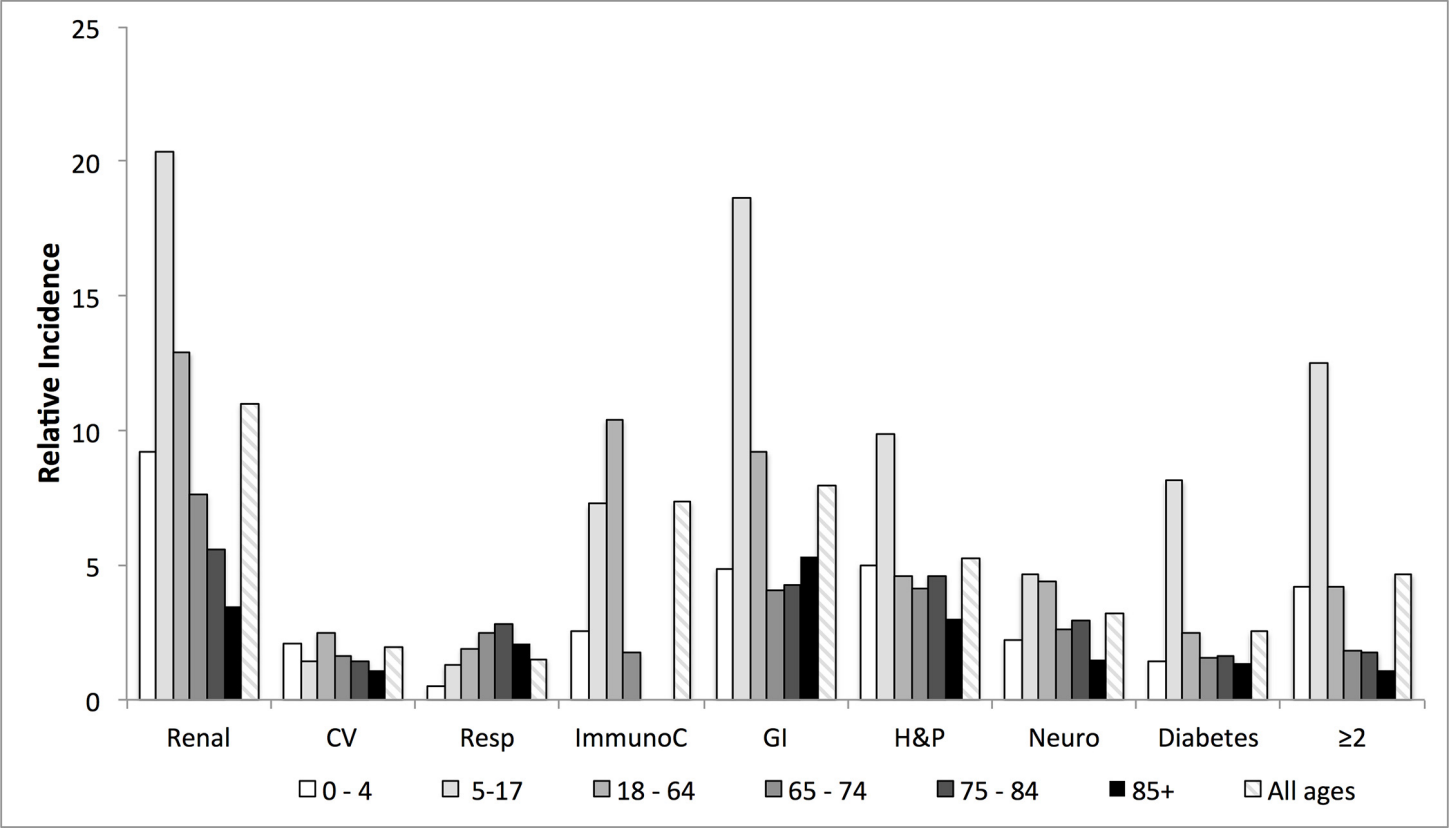
Incidence rate ratio of NGE hospitalizations among eight risk groups, compared to the healthy population (mean incidence, 2002–2013). CV = cardiovascular, Resp = respiratory, ImmunoC = Immunocompromising, GI = gastrointestinal, H&P = Hepatic and pancreatic, Neuro = neurological conditions, ≥ 2 = having 2 or more chronic conditions.

Considering each age category, NGE-attributable hospitalization rates in patients with a chronic medical condition were higher in all age groups and for all chronic condition groups, relative to the healthy population without a chronic condition (Table 5). The highest relative increases in NGE-attributable hospitalizations were observed in the adolescent (5–17 year) and adult (18–64 year) age groups (Fig 2). In these age groups, hospitalization rates for some chronic conditions were as high or higher as those seen in the overall elderly adult population (Table 5).

**Table 5:** Mean annual rate (per 10,000 person-years with 95% confidence intervals) of norovirus gastroenteritis (NGE)-attributable emergency department (ED) visits, hospitalizations and outpatient visits by age (2002–2013 MarketScan database).

Age (years)	All	Free of chronic condition	Persons with one chronic condition								
			Renal	Cardiovascular	Respiratory	ImmunoC	Gastrointestinal	Hep& Panc	Neurological	Diabetes	≥ 2 conditions
**NGE-attributable ED visits**											
0–4	80.0	84.3	127.4	100.8	39.3	90.6	108.5	124.6	102.4	87.7	115.3
	(75.1–89.7)	(77.8–95.8)	(124.8–173)	(96.8–134.7)	(24.6–62.5)	(87.3–122.3)	(101.8–125.7)	(117.4–158.2)	(96.3–115.1)	(83.7–116.4)	(107.5–132.6)
5–17	14.8	12.3	42.6	24.6	16.4	31.3	35.6	26.7	26.4	55.8	32.4
	(13.7–16.8)	(11.0–14.2)	(40.0–53.1)	(23.4–31.5)	(10.6–24.8)	(29.0–39.0)	(33.4–40.4)	(24.6–32.0)	(24.7–29.8)	(52.4–64.1)	(30.0–37.0)
18–64	14.7	12.6	15.9	6.6	12.1	21.2	11.5	12.5	12.5	9.1	9.3
	(13.6–16.8)	(11.4–14.6)	(14.8–18.2)	(6.1–7.5)	(7.5–18.5)	(20.0–25.3)	(10.4–13.1)	(11.6–14.3)	(11.6–14.1)	(8.4–10.3)	(8.5–10.7)
65–74	12.9	7.6	14.8	8.6	14.2	23.8	17.6	19.5	15.4	10.6	7.5
	(11.9–14.5)	(6.9–8.9)	(14.0–17.8)	(8.0–9.6)	(9.7–21.2)	(14.6–35.0)	(16.6–20.0)	(18.2–22.3)	(14.4–17.9)	(10.0–11.8)	(6.9–8.4)
75–84	18.9	12.6	17.5	13.1	20.1	0.0	28.1	24.5	26.1	16.9	10.3
	(17.5–21.5)	(11.5–14.4)	(16.4–20.6)	(12.1–14.9)	(13.4–31.1)	(0.0–93.7)	(26.4–31.4)	(22.9–28.9)	(24.4–29.7)	(15.8–19.1)	(9.3–11.7)
85+	33.5	19.6	22.7	23.9	33.5	0.0	65.0	33.9	26.5	24.5	17.9
	(31.0–37.8)	(18.1–22.6)	(22.0–28.7)	(22.2–27.1)	(21.5–55.2)	(0.0–534.7)	(61.2–74.6)	(32.6–46.0)	(25.0–31.9)	(23.1–29.2)	(16.6–20.1)
**NGE-attributable hospitalizations**											
0–4	14.2	15.8	145.2	32.9	7.9	40.2	76.7	79.0	34.8	22.2	66.5
	(13.4–15.6)	(14.8–17.3)	(135.4–178.2)	(31.6–44.8)	(5.0–11.4)	(32.8–48.5)	(71.8–88.6)	(75.1–102.7)	(32.9–41.6)	(17.4–27.5)	(61.7–76)
5–17	1.4	1.5	30.5	2.1	1.9	10.9	28.0	14.8	7.0	12.2	18.8
	(1.3–2.6)	(1.3–1.5)	(29.1–38.1)	(1.6–2.7)	(1.3–2.8)	(10.6–15.4)	(26.1–31.7)	(14.0–17.9)	(6.5–8.0)	(11.5–14.5)	(17.5–21.2)
18–64	1.9	1.0	12.9	2.5	1.9	10.4	9.2	4.6	4.4	2.5	4.2
	(1.7–2.1)	(0.9–1.1)	(11.9–14.6)	(2.3–2.7)	(1.5–2.9)	(9.9–12.8)	(8.4–10.2)	(4.3–5.1)	(4.0–4.9)	(2.2–2.7)	(3.9–4.7)
65–74	5.5	3.1	23.6	5.1	7.7	5.4	12.6	12.8	8.1	4.8	5.6
	(5.0–6.2)	(2.8–3.5)	(22.3–26.8)	(4.7–5.6)	(5.1–11.5)	(5.3–14.5)	(11.8–14.4)	(12.0–14.6)	(7.6–9.5)	(4.5–5.4)	(5.2–6.2)
75–84	10.5	4.8	26.7	7.0	13.4	0.0	20.5	22.1	14.1	7.7	8.5
	(9.6–11.7)	(4.4–5.4)	(25.1–30.7)	(6.5–7.9)	(9.0–20.5)	(0.0–255.4)	(19.1–23.3)	(20.9–25.4)	(13.2–16.4)	(7.3–8.8)	(7.9–9.5)
85+	21.3	11.8	40.6	12.8	25.0	NA	62.8	35.9	18.0	16.3	13.2
	(19.6–23.7)	(10.8–13.5)	(38.6–48.0)	(11.9–14.7)	(16.0–39.9)	NA	(58.7–72.3)	(34.6–44.4)	(16.9–21.0)	(15.1–19.6)	(12.1–14.8)
**NGE-attributable Outpatient visits**											
0–4	354.7	386.3	680.1	526.0	165.7	571.5	514.1	531.6	280.0	397.8	287.5
	(328.6–398.4)	(353.1–438.2)	(633.9–793.3)	(490.0–599.8)	(152.7–186.6)	534.7–643.6)	(475.9–575.8)	(495.2–609.2)	(259.1–316.5	(366.8–455.3)	(264.6–323.2)
5–17	112.1	90.6	186.4	102.9	115.9	207.7	198.9	148.5	115.0	177.8	146.4
	(104.7–124.1)	(82.2–103.7)	(174.5–211.5)	(95.8–118.9)	(108.4–128.5)	(194.5–233.1)	(184.5–221.0)	(137.5–167.2)	(107.9–126.4)	(167.1–195.7)	(136.2–162.9)
18–64	40.4	33.6	42.4	25.9	33.3	95.0	81.5	43.3	33.5	30.7	39.0
	(37.2–45.5)	(30.3–38.8)	(39.1–47.6)	(24.0–29.0)	(30.7–37.2)	(87.9–107.0)	(75.2–92.1)	(40.0–47.9)	(31.0–37.4)	(28.4–34.8)	(36.1–42.9)
65–74	52.2	42.5	63.9	32.3	41.8	181.5	95.3	68.3	62.8	43.9	26.0
	(47.9–58.1)	(38.2–48.4)	(59.8–71.4)	(30.1–35.3)	(38.7–46.6)	(151.4–215.2)	(88.2–106.3)	(63.4–75.8)	(58.8–69.3)	(40.9–48.8)	(24.0–29.3)
75–84	62.6	61.1	79.1	42.5	70.1	127.2	128.8	112.4	70.7	66.1	33.9
	(57.1–69.9)	(55.8–68.9)	(59.8–71.4)	(39.1–47.8)	(65.0–78.22)	(93.1–165.2)	(119.3–144.4)	(104.2–126.2)	(65.4–80.0)	(61.6–75.4)	(30.9–38.2)
85+	96.7	76.0	108.8	64.1	96.4	0.0	198.7	186.5	111.6	98.4	48.9
	(89.2–108.7)	(69.4–85.9)	(101.1–124.4)	(59.4–71.0)	(89.7–107.9)	(0.0–569.0)	(184.6–223.1)	(174.8–209.3)	(104.4–124.1)	(92.6–110.4)	(45.1–54.5)

ImmunoC = immunocompromising condition, Hep & Panc = hepatic and pancreatic condition, ≥ 2 = having at least 2 chronic conditions

The total proportion of NGE hospitalizations across all age groups that occurred in the population with a chronic condition was 51.6%. In the different age groups the proportions were 14.3%, 43.4%, 51.7%, 71.7%, 67.5% and 85.5% among 0–4, 5–17, 18–64, 65–74, 75–84 and 85+ year olds, respectively. The most important contributing groups were patients with gastrointestinal conditions, and the group of patients with two or more chronic conditions. In these groups the proportion of NGE hospitalizations (all ages) was 19.9% and 7.3%, respectively. The proportion of NGE-attributable ED and outpatient visits occurring in the population with any chronic medical conditions across all age groups was 18.0% and 20.4%, respectively.

## Discussion

Our study is the first to assess the burden of NGE in individuals with chronic medical conditions. NGE-attributable hospitalizations were substantially higher in patients with a chronic condition compared to the total population. Higher rates of hospitalization, in the absence of increased rates of higher ED or outpatient department visit rates, may reflect more severe disease or a higher propensity to admit patients with underlying chronic conditions.

In contrast to other chronic conditions, patients with chronic gastrointestinal or immunocompromising conditions had higher rates of all health utilization outcomes for NGE. This finding is consistent with previous reports suggesting that NGE may be particularly severe or protracted in immunocompromised patients [4,13–15]. The interpretation of the estimates for persons with gastrointestinal conditions is more difficult. It is possible that recurring gastro-intestinal symptoms have been interpreted as infectious AGE, thereby artificially increasing the overall AGE rates and as a consequence also the norovirus-attributable AGE rates in these patients. Intriguingly, the highest incidence ratio for norovirus-attributable hospitalizations (28.0 per 10,000 person-years) was seen in the 5–17 year olds. The highest hospitalization rates for NGE were found in the group with a chronic renal condition, which may have included kidney transplant patients who are at high risk for severe NGE [6]. More in-depth analyses of the association between chronic gastrointestinal or renal conditions and norovirus are needed to better understand these observations.

Several recent modelling studies have shown the substantial burden caused by NGE in terms of hospitalizations, deaths and ambulatory visits in the US and countries in Europe [7,10,11]. Here we highlight that NGE patients with a variety of chronic medical conditions have higher rates of healthcare utilization, particularly hospitalization, than those without chronic conditions. The main strengths of our study include the use of a large, nationally representative database. The age distribution in MarketScan population is comparable to the general US population except for individuals aged 18 to 64 years owing to the fact that the database contains a greater proportion of the working population aged 18–64 years who are covered by employer-sponsored health plan [16]. The use of such a large population set allowed us to perform NGE disease estimates stratified by age and chronic condition. The only group for which we did not have sufficient cases to perform the analyses were the 75+ age groups with an immunocompromising condition. Furthermore, our findings are consistent with the known epidemiology of NGE: as observed by others, the model predicted higher rates of AGE and NGE-attributable episodes in winter, with the highest rates of ambulatory visits and hospitalizations among children 0–4 years and elderly 85+ years [10,11,17,18]. Increased rates of NGE-attributable hospitalization during the years 2002–2003 and 2006–2007 are consistent with the higher burden reported in these years due to the emergence of new norovirus strains [19,20].

For groups over 5 years of age, our age-specific estimates of NGE-attributable outpatient and ED visits for the overall population are comparable to those reported by Gastañaduy et al [11], who used the same database (2001–2009) and a similar model, although we did not include the 2001–2002 season. Similarly, our estimates of NGE-attributable hospitalizations for age groups over 5 years are comparable to Lopman et al, who used data from the National Inpatient Sample [10]. Compared to the studies of both Gastañaduy et al [11] and Lopman et al [10] our estimates are higher, however, for 0–4 year olds, with rates approximately 2-fold higher. These differences may be related to differences in the study years or the data source. Our estimates are also comparable to those obtained in a prospective study in children using the New Vaccine Surveillance Network in the US [21]. Is this study, the average rates of hospitalization, ED visits, and outpatient visits for NGE were 7.2, 140.5, and 318.3 per 10,000 children younger than 5 in the period October 2008—September 2010. In the current study, the average rates in the period July 2008—June 2010 were 5.0, 42.5 and 288.0 per 10,000 children younger than 5 for the same respective outcomes. The lower rates obtained in the current study for ED visits may be related to the fact that patients who move from the ED to a hospital ward are possibly coded only as hospitalizations, resulting in an underestimate of ED visits.

The causal agent in cases of AGE is infrequently diagnosed, which means that direct estimates of the disease burden using laboratory-confirmed cases are likely to be substantial underestimates. While indirect modelling techniques overcome this limitation in the data, the present model relies on the assumption that NGE makes up the vast majority of AGE when an etiologic agent was not identified, leading to possible over-estimation of the disease burden. Other potential limitations include the inability of the model to control for seasonal factors that may have influenced health care utilization behaviors, such as holiday periods, the assumption that ICD-coding practices remained constant or that the propensity of patients to seek care or be hospitalized did not change over the study period, and changes in trends for certain etiological agents such as decreases in rotavirus hospitalizations following the introduction of rotavirus vaccination in 2009. In addition, our use of a database based on insurance claims automatically excludes the uninsured, thereby limiting the extrapolation to the entire US population. In the present study, when comparing the rates among different risk groups, these limitations may be less important as they can be expected to occur similarly across the different groups.

We identified pre-existing chronic medical conditions exclusively based on ICD-9 codes. We cannot be sure that every subject classified as having a chronic condition was actively suffering from the condition. Reassuringly, our estimated rates of chronic conditions are very comparable to previous reports for the US population, although such comparisons are hampered by differences in classification of chronic conditions and prevalence estimation methods. In a recent analysis of the prevalence of multiple chronic conditions among US adults, Ward et al found 24.3% of noninstitutionalized US adults to have only 1 chronic condition and 25.5.1% to have more than 1 [22]. In our dataset, 24.9% and 20.9% of adults had 1 or more than 1 chronic condition, respectively, during the study period. We included a large set of codes, based on a consensus between the authors, which may have led to the inclusion of subjects with an acute self-limiting condition rather than a truly chronic condition. Both of these limitations would have theoretically led to a dilution of the effect that we observed and we therefore believe they do not invalidate our findings.

We observed the highest relative risk estimates for NGE-associated hospitalizations among 5–17 and 18–64 year olds with chronic medical conditions, as compared with the same age groups in the healthy population. This is not totally unexpected as hospitalization rates in healthy 5–17 or 18–64 year olds can be expected to be relatively low, whereas hospitalization rates among healthy small children and elderly will be relatively high already. Similar observations have been made for populations at high versus low risk of influenza complications, in which the increase in hospitalization outcomes was proportionally higher in at-risk 18–64 year olds than in the at-risk elderly [23]. The practical consequence of our observation is that the biggest gain from targeting subjects with chronic medical conditions for prevention can be expected among these younger groups. Among the 5–17 year olds for example, preventing norovirus disease in the 18% with underlying medical conditions would avoid 43% of all norovirus-associated hospitalizations in this age group. Among 85+ year-olds, preventing norovirus disease in the 89% with underlying medical conditions would avoid an equal proportion (89%) of all norovirus-associated hospitalizations, not justifying the additional effort of identifying these high risk populations.

Development of norovirus vaccines is underway with data from a Phase 1 clinical study showing a candidate bivalent VLP norovirus vaccine to be immunogenic and reducing the severity of norovirus disease in a human challenge model [24,25]. Studies to identify patient groups either at high risk of complicated NGE or who have high rates of utilization of health services may help to inform future vaccination policy. Along with other studies of the NGE burden, our study indicates that NGE hospitalizations (an indicator of disease severity) are highest in the very young and elderly. In addition, our study highlights that the burden of NGE hospitalization in individuals of all ages with a chronic illness is substantially higher than the general population rates.

## Conclusion

The presence of a chronic pre-existing medical condition leads to higher rates of health care utilization due to NGE. A disproportionate segment (52%) of all norovirus hospitalizations occurred in the 37% of the population that had at least one chronic medical condition, who along with children under 5 and elderly adults, may merit special attention in NGE prevention strategies. The use of modeling techniques to derive norovirus specific AGE rates and the reliance upon coded diagnoses suggest caution in interpreting our results pending further confirmation.

## Supporting Information

S1 Fig Rates of acute gastroenteritis (AGE) and acute gastroenteritis attributed to norovirus (NGE) leading to emergency department visits, outpatient visits and hospitalizations, per 100,000 person-years, (MarketScan database, from 01 July until 30 June 2002 to 2013). (TIFF)

S1 Table Demographic features of the total population in the MarketScan database (01 July 2002–30 June 2013). *includes subjects with only one chronic condition. ImmunoC = immunocompromising condition, Hep & Panc = hepatic and pancreatic condition, ≥ 2 = having at least 2 chronic conditions.
(DOCX)

## References

[1] Lopman BA, Steele D, Kirkwood CD, Parashar UD. The Vast and Varied Global Burden of Norovirus: Prospects for Prevention and Control. PLoS Med. 2016;13: e1001999 10.1371/journal.pmed.1001999 27115709 PMC4846155

[2] Koo HL, Ajami N, Atmar RL, DuPont HL. Noroviruses: The leading cause of gastroenteritis worldwide. Discov Med. 2010;10: 61–70. 20670600 PMC3150746

[3] Centers for Disease Control and Prevention. Updated norovirus outbreak management and disease prevention guidelines. MMWR Recomm Rep. 2011;60: 1–18. 21368741

[4] Koo HL, DuPont HL. Noroviruses as a potential cause of protracted and lethal disease in immunocompromised patients. Clin Infect Dis. 2009;49: 1069–1071. 10.1086/605558 19705972

[5] Lopman BA, Reacher MH, Vipond IB, Sarangi J, Brown DWG. Clinical manifestation of norovirus gastroenteritis in health care settings. Clin Infect Dis. 2004;39: 318–324. 15306997 10.1086/421948

[6] Mattner F, Sohr D, Heim A, Gastmeier P, Vennema H, Koopmans Met al. Risk groups for clinical complications of norovirus infections: an outbreak investigation. Clin Microbiol Infect. 2006;12: 69–74. 16460549 10.1111/j.1469-0691.2005.01299.x

[7] Haustein T, Harris JP, Pebody R, Lopman BA. Hospital admissions due to norovirus in adult and elderly patients in England. Clin Infect Dis. 2009;49: 1890–1892. 10.1086/648440 19911997

[8] Harris JP, Edmunds WJ, Pebody R, Brown DW, Lopman BA. Deaths from norovirus among the elderly, England and Wales. Emerging Infect Dis. 2008;14: 1546–1552. 10.3201/eid1410.080188 18826817 PMC2609872

[9] Markov PV, Crowcroft NS. Modelling the unidentified mortality burden from thirteen infectious pathogenic microorganisms in infants. Epidemiol Infect. 2007;135: 17–26. 16740187 10.1017/S0950268806006625 PMC2870553

[10] Lopman BA, Hall AJ, Curns AT, Parashar UD. Increasing rates of gastroenteritis hospital discharges in US adults and the contribution of norovirus, 1996–2007. Clin Infect Dis. 2011;52: 466–474. 10.1093/cid/ciq163 21258098

[11] Gastañaduy PA, Hall AJ, Curns AT, Parashar UD, Lopman BA. Burden of norovirus gastroenteritis in the ambulatory setting—United States, 2001–2009. J Infect Dis. 2013;207: 1058–1065. 10.1093/infdis/jis942 23300161

[12] Fiore AE, Shay DK, Broder K, Iskander JK, Uyeki TM, Mootrey G, et al Prevention and control of seasonal influenza with vaccines: recommendations of the Advisory Committee on Immunization Practices (ACIP), 2009. MMWR Recomm Rep. 2009;58: 1–52. 19644442

[13] Roddie C, Paul JPV, Benjamin R, Gallimore CI, Xerry J, Gray JJ, et al Allogeneic hematopoietic stem cell transplantation and norovirus gastroenteritis: a previously unrecognized cause of morbidity. Clin Infect Dis. 2009;49: 1061–1068. 10.1086/605557 19705974

[14] Siebenga JJ, Beersma MFC, Vennema H, van Biezen P, Hartwig NJ, Koopmans Met al. High prevalence of prolonged norovirus shedding and illness among hospitalized patients: a model for in vivo molecular evolution. J Infect Dis. 2008;198: 994–1001. 10.1086/591627 18774885

[15] Simon A, Schildgen O, Maria Eis-Hübinger A, Hasan C, Bode U, Buderus S, et al Norovirus outbreak in a pediatric oncology unit. Scand J Gastroenterol. 2006;41: 693–699. 16716968 10.1080/00365520500421694

[16] Karve S, Krishnarajah G, Korsnes JS, Cassidy A, Candrilli SD. Burden of acute gastroenteritis, norovirus and rotavirus in a managed care population. Hum Vaccin Immunother. 2014;10: 1544–1556. 10.4161/hv.28704 24732307 PMC5396247

[17] Phillips G, Tam CC, Conti S, Rodrigues LC, Brown D, Iturriza-Gomara M, et al Community incidence of norovirus-associated infectious intestinal disease in England: improved estimates using viral load for norovirus diagnosis. Am J Epidemiol. 2010;171: 1014–1022. 10.1093/aje/kwq021 20360244

[18] Bernard H, Höhne M, Niendorf S, Altmann D, Stark K. Epidemiology of norovirus gastroenteritis in Germany 2001–2009: eight seasons of routine surveillance. Epidemiol Infect. 2014;142: 63–74. 10.1017/S0950268813000435 23517686 PMC9152553

[19] Centers for Disease Control and Prevention (CDC). Norovirus activity—United States, 2006–2007. MMWR Morb Mortal Wkly Rep. 2007;56: 842–846. 17717513

[20] Centers for Disease Control and Prevention (CDC). Norovirus activity—United States, 2002. MMWR Morb Mortal Wkly Rep. 2003;52: 41–45. 12570319

[21] Payne DC, Vinjé J, Szilagyi PG, Edwards KM, Staat MA, Weinberg GA, et al Norovirus and medically attended gastroenteritis in U.S. children. N Engl J Med. 2013;368: 1121–1130. 10.1056/NEJMsa1206589 23514289 PMC4618551

[22] Ward BW, Schiller JS, Goodman RA. Multiple chronic conditions among US adults: a 2012 update. Prev Chronic Dis. 2014;11: E62 10.5888/pcd11.130389 24742395 PMC3992293

[23] Mullooly JP, Bridges CB, Thompson WW, Chen J, Weintraub E, Jackson LA, et al Influenza- and RSV-associated hospitalizations among adults. Vaccine. 2007;25: 846–855. 17074423 10.1016/j.vaccine.2006.09.041

[24] Bernstein DI, Atmar RL, Lyon GM, Treanor JJ, Chen WH, Jiang X, et al Norovirus vaccine against experimental human GII.4 virus illness: a challenge study in healthy adults. J Infect Dis. 2015;211: 870–878. 25210140 10.1093/infdis/jiu497 PMC5914500

[25] Treanor JJ, Atmar RL, Frey SE, Gormley R, Chen WH, Ferreira J, et al A novel intramuscular bivalent norovirus virus-like particle vaccine candidate—reactogenicity, safety, and immunogenicity in a phase 1 trial in healthy adults. J Infect Dis. 2014;210: 1763–1771. 10.1093/infdis/jiu337 24951828 PMC8483568

