# Supplementary figures and images for: Modelling Estimates of Norovirus Disease in Patients with Chronic Medical Conditions
Source: PLoS One. 2016 Jul 20;11(7):e0158822. doi: 10.1371/journal.pone.0158822 (PMC4954678; doi:10.1371/journal.pone.0158822)

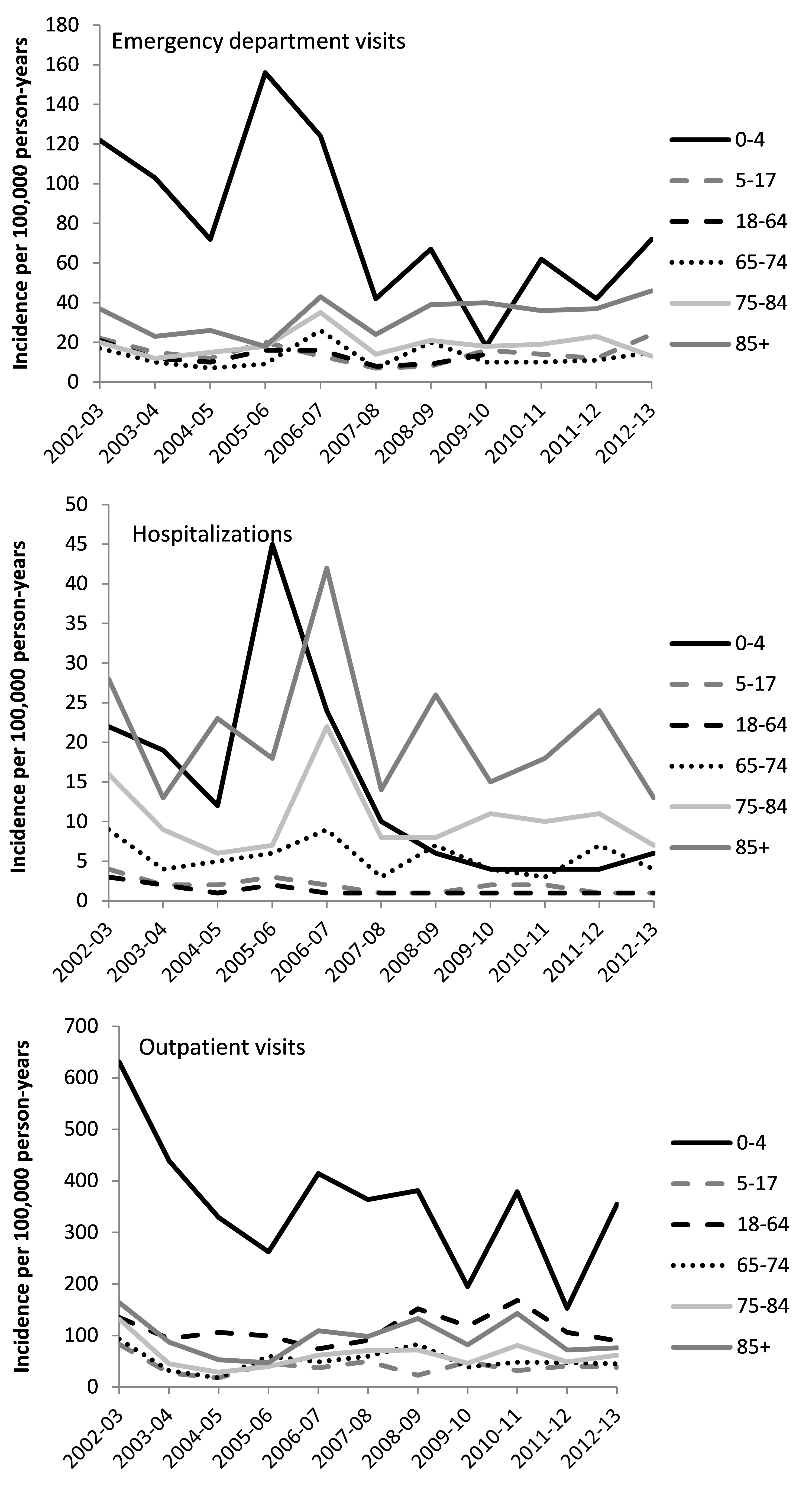

Supplement: S1 Fig — (TIFF) [file pone.0158822.s001.tiff]
