# Supplementary material for: Modelling Estimates of Norovirus Disease in Patients with Chronic Medical Conditions
Source: PLoS One. 2016 Jul 20;11(7):e0158822. doi: 10.1371/journal.pone.0158822 (PMC4954678; doi:10.1371/journal.pone.0158822)
Supplement: S1 Table — *includes subjects with only one chronic condition. ImmunoC = immunocompromising condition, Hep & Panc = hepatic and pancreatic condition, ≥ 2 = having at least 2 chronic conditions. (DOCX) [file pone.0158822.s002.docx]

**Supporting information**

**S1 Table: Demographic features of the study population (MarketScan database, 01 July 2002 – 30 June 2013).**

|  |  | **Total population in the MarketScan database n(%)** | **Population without a chronic condition n(%)** | **Population with a chronic condition n(%)*** | | | | | | | | |
| --- | --- | --- | --- | --- | --- | --- | --- | --- | --- | --- | --- | --- |
|  |  |  |  | **Renal** | **Cardiovascular** | **Respiratory** | **ImmunoC** | **Gastrointestinal** | **Hep& Panc** | **Neurological** | **Diabetes** | **≥ 2 conditions** |
| All |  | 9,028,466 (100) | 5,733,147 (100) | 38,316 (100) | 225,989 (100) | 570,461 (100) | 9,336 (100) | 503,082 (100) | 211,379 (100) | 108,099 (100) | 282,044 (100) | 1,346,583 (100) |
| Gender | Male | 3,935,424 (43.6) | 2,603,841 (45.4) | 17,850 (46.6) | 107,906 (47.8) | 266,847 (46.8) | 3,986 (42.7) | 183,113 (36.4) | 61,875 (29.3) | 40,798 (37.7) | 120,796 (42.8) | 528,412 (39.2) |
|  | Female | 5,093,042 (56.4) | 3,129,336 (54.6) | 20,466 (53.4) | 118,083 (52.2) | 303,614 (53.2) | 5,350 (57.3) | 319,969 (63.6) | 149,504 (70.7) | 67,301 (62.3) | 161,248 (57.2) | 818,171 (60.8) |
| Age (yrs) | 0-4 | 1,435,556 (15.9) | 1,245,319 (21.7) | 933 (2.4) | 2,271 (1.0) | 149,659 (26.2) | 2,028 (21.7) | 9,401 (1.9) | 1,402 (0.7) | 10,532 (9.7) | 1,067 (0.4) | 12,944 (1.0) |
|  | 5-17 | 1,308,992 (14.5) | 1,079,875 (18.8) | 1,608 (4.2) | 1,411 (0.6) | 147,917 (25.9) | 1,686 (18.1) | 24,545 (4.9) | 7081 (3.3) | 15,089 (14.0) | 6,657 (2.4) | 23,123 (1.7) |
|  | 18-64 | 5,368,201 (59.5) | 3,245,390 (56.6) | 25,780 (67.6) | 138,595 (61.3) | 246,303 (43.2) | 5,315 (56.9) | 424,664 (84.4) | 190,181 (90.0) | 68,415 (63.3) | 236,967 (84.0) | 786,485 (58.4) |
|  | 65-74 | 411,513 (4.6) | 97,069 (1.7) | 3,802 (9.9) | 308,870 (13.7) | 14,409 (2.5) | 230 (2.5) | 27,591 (5.5) | 7,758 (3.7) | 5,012 (4.6) | 23,337 (8.3) | 201,418 (15.0) |
|  | 75-84 | 344,768 (3.8) | 47,454 (0.8) | 3,751 (9.8) | 32,576 (14.4) | 8,910 (1.6) | 67 (0.7) | 13,510 (2.7) | 3,769 (1.8) | 5,528 (5.1) | 10,980 (3.9) | 218,223 (16.2) |
|  | 85+ | 159,436 (1.7) | 18,070 (0.3) | 2,336 (6.1) | 20,249 (9.0) | 3,263 (0.6) | 10 (0.1) | 3,371 (0.7) | 1,188 (0.6) | 3,523 (3.3) | 3,036 (1.1) | 104,390 (7.8) |

*includes subjects with only one chronic condition. ImmunoC= immunocompromising condition, Hep & Panc = hepatic and pancreatic condition, ≥ 2 = having at least 2 chronic conditions
